# Effects of oral selenium and magnesium co-supplementation on lipid metabolism, antioxidative status, histopathological lesions, and related gene expression in rats fed a high-fat diet

**DOI:** 10.1186/s12944-018-0815-4

**Published:** 2018-07-21

**Authors:** Qian Zhang, Zhi-Yong Qian, Peng-Hui Zhou, Xiao-li Zhou, Da-Long Zhang, Ning He, Jing Zhang, Ying-Hua Liu, Qing Gu

**Affiliations:** 1 Department of Toxicology, Tianjin Centers for Disease Control and Prevention, No. 6, Huayue Road, Hedong District, Tianjin, 300011 People’s Republic of China; 2 Infectious Diseases Control Institute, Tianjin Centers for Disease Control and Prevention, No. 6, Huayue Road, Hedong District, Tianjin, 300011 People’s Republic of China

**Keywords:** Hyperlipidemia, Selenium, Magnesium, Lipid profile, High-fat, mRNA expression

## Abstract

**Background:**

Supplementation with Selenium (Se) has been shown to lower blood cholesterol and increase tissue concentrations of the antioxidant glutathione (GSH); however, the effects of Se supplementation, in combination with supplemental magnesium, on high fat-induced hyperlipidemia have not been studied. This study was designed to elucidate the effects of oral selenium and magnesium co-supplementation on antihyperlipidemic and hepatoprotective, antioxidative activities, and related gene expression in a hyperlipidemic rat model.

**Methods:**

Forty male Sprague Dawley rats were divided into 4 groups: one group served as control group (CT), provided control diet; The other groups were made hyperlipidemic with high-fat diet; specifically, a high-fat diet group (HF); low-dose selenium (0.05 mg/kg·bw) + low-dose magnesium (5.83 mg/kg·bw) supplement high-fat diet group (HF + LSe + LMg) and high-dose selenium (0.10 mg/kg·bw) + high-dose magnesium (58.33 mg/kg·bw) supplement high-fat diet group (HF + HSe + HMg). The first 4 weeks of the experiment was a hyperlipidemia inducing period using high-fat diet and the following 8 weeks involved in selenium and magnesium co-supplementation. On day 0, 20, 40 and 60 of the intervention, lipid profile was measured. At the end of the 12-week experiments, final blood and liver samples were collected for the measurements of lipid profile, antioxidative indexes, pathological examination, and liver lipid metabolism related gene expression.

**Results:**

The elevated levels of serum and liver total cholesterol (TC) and serum LDL-C induced by feeding high-fat diets were significantly reduced by low-dose Se and Mg co-supplementation. Both doses of selenium and magnesium co-supplementation notably decreased the blood and liver TG levels, liver function indexes ALT and AST and the ratio of TC/HDL-C and TG/HDL-C. In contrast, Se and Mg supplementation showed a substantial increase in Se-dependent glutathione peroxidase (GSH-Px) and SOD activities and an significant reduce of level of MDA of hyperlipidemic rats. Oil Red O staining showed that selenium and magnesium co-supplementation significantly reduced hepatic intracellular triacylglycerol accumulation. H&E staining also showed that selenium and magnesium co-supplementation can attenuate liver steatosis. Selenium and magnesium co-supplementation remarkably inhibited the mRNA expression level of hepatic lipogenesis genes liver X receptor alpha (LXRα),SREBP-1c and FASN (fatty acid synthase), regulated the mRNA expression levels of liver enzymes related to cholesterol metabolism, including the down regulation of 3-hydroxy-3-methylglutaryl coenzyme A reductase (HMGR) and the upregulation of cholesterol 7α-hydroxylase (CYP7A1) and lecithin cholesterol acyltransferase (LCAT) in the liver of hyperlipidemia rats.

**Conclusions:**

Oral selenium and magnesium co-supplementation inhibited an increase of lipid and liver profile and liver function index induced by a high-fat diet, and enhanced the activity of the antioxidant enzymes. Selenium combined with magnesium is a promising therapeutic strategy with lipid-lowering and antioxidative effects that protects the liver against hyperlipidemia.

## Background

Hyperlipidemia is a lipid metabolism disorder that causes abnormally elevated levels of cholesterol (TC), triglycerides (TG) and lipoproteins in the blood. Hyperlipidemia is widely accepted to be a key risk factor for atherosclerosis, coronary heart disease (CHD) and peripheral vascular disease [1]. Statins are widely used in clinical practice to reduce low-density lipoprotein cholesterol levels and decreasing cardiovascular events. However, because of the high prices and intolerance, side effects or, simply, patient preference, it is more and more impending to explore more effective and safer alternative therapies to hyperlipidemia. Nutraceuticals and functional food ingredients that are beneficial to vascular health may represent useful compounds that are able to reduce the overall cardiovascular risk induced by dyslipidaemia by acting parallel to statins or as adjuvants in case of failure or in situations where statins cannot be used [1, 2].

Magnesium (Mg) is an essential cation which can be adjusted to rapidly effect atherosclerosis. Magnesium intake is negatively related to risk of CVD and CVD-related mortality [3]. There is substantial evidence to demonstrate the relationship between magnesium intake and oxidative, metabolic and inflammatory disorders [4–6]. Several studies have revealed that Selenium (Se) has beneficial effects on DNA damage repair, antioxidant properties, liver and kidney protection, cancer prevention, diabetes and metabolic syndrome [7–11]. Our previous data showed that selenium (Se) is involved in the lipid metabolism, since selenium-enriched yeast-treated high fat-fed rats had reduced blood and hepatic lipids levels, atherogenic index (AI), by decreasing the mRNA expression of HMGR [12]. A survey of the literature shows that while the known activities of Mg or Se had led to real hopes for them to be therapies of dyslipidemia, up to now, there has not been a report on the effect of combined selenium and magnesium intervention on hyperlipidemia caused by high fat diet. Clear therapeutic benefit in this pathology is presently doubtful. We have hypothesized that adequate supplementation of selenium plus magnesium, as a potential nutraceutical, may alleviate the hyperlipidemic and hepatic effects of a high-fat diet. In this study, high fat-fed rats have received oral supplementation of sodium selenite and magnesium gluconate to assess the effect on the level of lipid profile, oxidative stress and related gene expression.

## Materials and methods

### Animals, diets and experimental design

The experiment was conducted on 40 adult male Sprague Dawley (SD) rats, supplied by the Vital River Laboratory Animal Ltd. (Beijing, China). The rats, body weight of 200 ± 20 g, were housed at a constant temperature (23 ± 2 °C) and humidity (55 ± 5%) with a 12 h light/dark cycle (7:00 a.m. to 7:00 p.m.). Food and water were supplied ad libitum. Rats were individually housed in cages and assigned into two different dietary groups. After 1-week adaptive period on a basal diet, the 40 rats were randomly selected and assigned to four groups of 10 rats each, viz. (I) control group (CT), (II) high-fat diet fed group (HF/model group), (III) high-fat diet fed + low-dose sodium selenite (Se content: 0.05 mg/kg·bw) and low-dose magnesium gluconate (Mg^2+^ content: 5.83 mg/kg·bw) supplemented (HF + LSe + LMg), (IV) HFD fed + high-dose sodium selenite (Se content: 0.10 mg/kg·bw) and high-dose magnesium gluconate (Mg content: 58.33 mg/kg·bw) supplemented (HF + HSe + HMg). Sodium selenite was purchased from Sigma Aldrich Ltd. Magnesium gluconate was supplied by Beijing Huikangyuan Biotechnology Co., Ltd. (Beijing, China). On 0 to 4 weeks of experiment period, rats in CT group were given basal diet, and the other groups of animals was fed on a high-fat diet (63.6% standard diet, 20% sucrose, 15% lard, 1.2% cholesterol and 0.2% cholic acid sodium) to generate a diet-induced hyperlipidemia model as previously reported [13]. Composition (%) of basal and high-fat chow is shown in Table 1. After 4 weeks, rats were given sodium selenite and magnesium gluconate solution by oral gavage for 8 weeks. CT and HF groups had no selenium or magnesium supplementation but an equivalent amount of distilled water by the same route. The rats were fed for 12 weeks. Throughout the 8-weeks intervention period, body weight was obtained weekly and at the time of culling. Food consumption (energy intake) of the animals was measured weekly. On day 0, 20, 40 and 60 of the Se/Mg intervention, whole blood was collected from the eye canthus in each animal under general anesthesia and serum for the determination of total cholesterol (TC), triglyceride (TG), low density lipoprotein cholesterol (LDL-C) and high density lipoprotein cholesterol (HDL-C) concentrations. At the end of the experiment, rats in all four groups were fasted for 12 h and blood samples were taken from the abdominal aorta under anesthesia. The livers were rapidly dissected, frozen in liquid nitrogen, and stored at − 80 °C until analysis.

**Table 1:** General composition of the experimental diets

Component	Control diet^A^	High-fat diet ^B^
Crude ash (%)	9.3	4.0
Crude protein (%)	16.0	9.91
Crude fat (%)	2.9	17.0
Crude fiber (%)	5.8	5.5
Total phosphorus (%)	0.66	0.54
Calcium (%)	1.83	0.73
Moisture (%)	9.39	7.30
Selenium (mg/kg)	0.169	0.131
Magnesium (mg/kg)	2100	1400

All diet were prepared by HFK BIOSCIENCE Co., Ltd.(Beijing, China)

^A^Control diet provided to CT (ad libitum), HF, HF + LSe + LMg and HF + HSe + HMg groups during the acclimatization period. ^B^ High-fat diet provided to HF, HF + LSe + LMg and HF + HSe + HMg groups

### Doses of selenium

The Recommended Nutrient Intakes (RNI) for selenium is 60 μg/day for man and women. The tolerable upper intake level (UL) for adults is set at 400 μg/day based on selenosis as the adverse effect [14]. However, therapeutic, antioxidant and antitumourigenic effects in experimental studies have been consistently associated with supranutritional intakes of selenium, several times higher than the dose required to prevent clinical signs of Se deficiency, for anticancerogenic effect it is 100–500 μg Se/kg body weight/day (for rodents) [15]. Mice of selenium modified exopolysaccharide of *Lachnum* sp. (SeLEP-1b) fed with high-fat diet were given 50, 100, 200 mg/kg, respectively (the amount of selenium in SeLEP-1b was about 3.6 mg/g), SeLEP-1b (200 mg/kg) notably reduced the serum and liver lipids, atherogenic index, and enhanced the activities of antioxidant enzymes of hyperlipidemic mice [16]. Administration of the selenium in drinking water (20 μM Se) with a dose of 70 μg Se/rat/day for 16 weeks, increased the activity of superoxide dismutase in liver and serves as a useful protective therapy against damage induced by the thioacetamide. Administration of selenium in the dose 10 μg Se/kg bodyweight/day for 60 days prevented alcohol-related injuries to the testis of rats [17]. Therefore, the dosages of Se in the present study were similarly or higher than the dosages used previously in preventive studies, that was rats received 50 μg Se/kg bodyweight/day or 100 μg Se/kg bodyweight/day by intragastric administration.

### Doses of magnesium

Choi et al. [18] have reported that Mg-CUD (composed of 45.6% of Ursodeoxycholic acid, 45.6% of chenodeoxycholic acid, and 8.8% of magnesium) at 62.5 mg/kg (Mg content: 5.5 mg/kg) was selected as the optimal effective dose for evaluating the molecular mechanisms of Mg-CUD against D-Galactosamine-induced hepatotoxicity in rats. Adequate intake (AI) of 350 mg Mg/person per day (5.83 mg/kg body weight/day) is recommended for human [14]. In our study, everyday rats were administrated with magnesium by gavage and so they got an approx. 5.83 mg Mg/kg (the recommended dose of the magnesium) or 58.3 mg Mg/kg (10 times of the recommended dose of the magnesium) of the body weight per day additionally.

### Ethics statement

The animal experimental protocol was approved by the Committee on the Ethics of Animal Experiments of Tianjin Centers for Disease Control and Prevention (permit number: TJCDC0111). This study was carried out in strict accordance with The People’s Republic of China Laboratory Animal Regulations.

### Determination of oxidation index in serum

At the end of the experiment, antioxidant enzymes including superoxide dismutase (SOD), glutathione-Px (GSH-Px), total antioxidative capacity (T-AOC) and malondialdehyde (MDA) were measured in the serum using a Varioskan Flash microplate reader (Thermo Scientific, America) followed manufacturer’s instruction. The corresponding SOD, GSH-Px, T-AOC and MDA detection kits were from Nanjing Jiancheng Bio-engineering Institute (Nanjing, China).

### Biochemistry analysis

At the end of the experiment, blood samples were taken by abdominal aorta under anesthesia by pentobarbital sodium intraperitoneal injection. Serum alanine aminotransferase (ALT), aspartate aminotransferase (AST), total protein (TP), albumin (ALB) and glucose (GLU) levels were determined using a TOSHIBA TBA-40FR Automatic Analyzer (Toshiba, Tokyo, Japan) and the corresponding reagents ALT, AST, TP, ALB and GLU were purchased from BioSino Bio-Technology & Science Inc. (Beijing, China).

### Assay for liver TC and TG

Liver tissue (0.3 g) was weighed into a glass tube and 2.7 mL cold saline solution (pH 7.4) was added. Then the glass tube was inserted into the ice water mixer to keep low temperature, fully grinding with pestle until fibrous substance can not be seen. The liver homogenate was centrifuged with 2000 r/min, 15 min, and the supernatant was collected. The levels of TC and TG in 10% liver homogenate were detected by automatic biochemical analyzer.

### Assay for serum lipid profile

Before the intervention period (day 0) and on day 20, 40 and 60 of the intervention period, the rats were deprived of food for 16 h, whole blood was collected from eye canthus in each animal under general anesthesia and serum for the determination lipid content. The serum was separated from the blood by centrifugation at 3500 rpm for 10 min. The serum lipid profile including TC, HDL-C, LDL-C and TG was performed on TOSHIBA TBA-40FR Automatic Analyzer (Toshiba, Tokyo, Japan) using the corresponding commercial TC, HDL-C, LDL-C and TG kits (BioSino Bio-Technology & Science Inc., Beijing, China) for each parameter. Serum atherogenic index (AI), TC/HDL-C, TG/HDL-C and LDL-C/HDL-C were later calculated.

### Histopathology of liver

One liver sample from each rat was fixed in 10% (volume/volume) formaldehyde, embedded in paraffin and stained with hematoxylin-eosin (HE). A second liver sample was sectioned frozen with Leica CM1900 freezing microtome (10 μm). The sections were picked up on a glass slide and stained with Oil Red O dye.

### RNA isolation and quantitative RT-PCR

Total RNA was extracted from liver using TRIzol (ComWin Biotech, Beijing, China) according to the manufacturer’s protocol and reverse-transcribed into cDNA using Transcriptor First Strand cDNA Synthesis Kit (Roche, USA). Real-time quantitative polymerase chain reaction (qPCR) was performed with a LightCycler® 480 (Roche Applied Science, USA) real-time PCR system using the SYBR Premix Ex Taq (Tli RNase H Plus) (Takara, Japan). Primers of the target genes were synthesized by Sangon Biotech (Shanghai, China). The primer sequences are listed in Table 2. Gene expression was determined by normalizing to glyceraldehyde-3-phosphate dehydrogenase (GAPDH; EC1.2.1.12) and calculating the 2^-∆∆Ct^.

**Table 2:** Primer sequences used in this study

Target genes	Forward (5’-3’)	Reverse (5’-3’)
GAPDH	GCAAGTTCAACGGCACAG	CGCCAGTAGACTCCACGAC
HMG-CoA-R	TGTGGGAACGGTGACACTTA	CTTCAAATTTTGGGCACTCA
LDLR	AGCCGATGCATTCCTGACTC	AGTTCATCCGAGCCATTTTCAC
CYP7A1	ACGTGGTTGGAAGAAGCG	GAATGTGGGCAGCGAGAA
ACAT	GCTGAAGTGAACTACCCCTT	GAGCCATGCCTCTAGTACCT
LCAT	CCCAAGGCTGAACTCAGTAACCA	CGGTAGCACAGCCAGTTTACCA
DGAT2	GAGACTACTTTCCCATCCAG	GGTATCCAAAGATATAGTTCCT
LXRα	ACAACCCTGGGAGTGAGA	TAGCATCCGTGGGAACAT
PPARγ	TTGATTTCTCCAGCATTTC	GCTCTACTTTGATCGCACT
PPARα	CCTGGCCTTCTAAACATAGG	TCCCTGCTCTCCTGTATGGG
FASN	CTGGACTCGCTCATGGGTG	CATTTCCTGAAGTTTCCGCAG

### Statistical analyses

All data were analyzed using the SPSS software computer program (version 17.0) for Windows (SPSS, Inc., Chicago, IL, USA). All data are expressed as means ± standard deviation (SD). Kolmogorov–Smirnov tests were used to examine the validity of normality for data with different treatments. One-way analysis of variance (ANOVA) followed by LSD’s post-hoc test were used to compare the normally distributed data and Kruskal–Wallis test followed by the Mann-Whitney test were used to compare the continuous variables that do not follow the normal distribution. Values of *P* < 0.05 were considered significant. All graphs were plotted using Graphpad Prism 5.0 software (GraphPad Software, Inc., San Diego, CA, USA).

## Results

### Energy intake, body weight

**Fig. 1:**
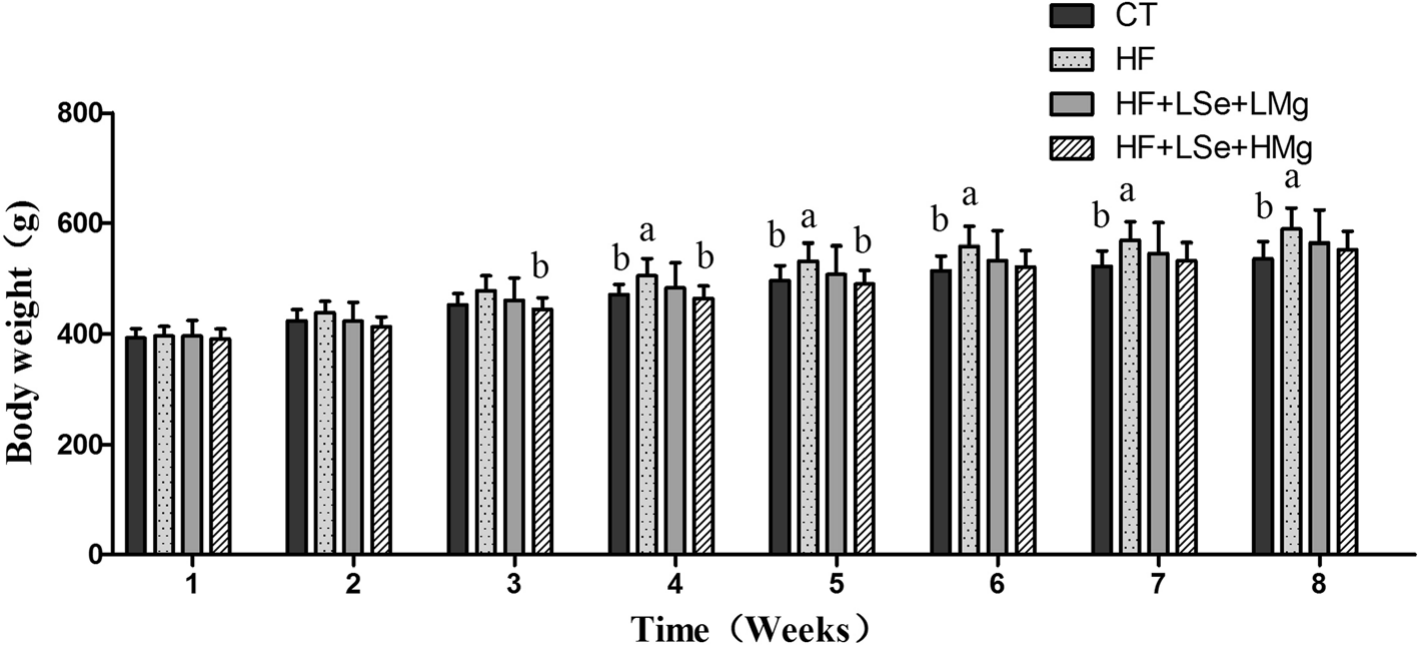
Effects of oral Se and Mg co administration on body weight in hyperlipidemia rats (*n* = 10, mean ± SD) a signify mean values that are significantly different from CT (*P* < 0.05). b signify mean values that are significantly different from HF (*P* < 0.05)

During the administration period, the initial body weight of the rats showed no significant difference between groups. After 4 weeks of the administration period, the rats in HF group exhibited the expected increasing trend in body weight compared to the control diet group. The body weight of rats in HF group on weeks 4 to 8 were higher than the CT group (*P* < 0.05).The body weight of rats in the HF + HSe + HMg group on weeks of 3 to 5 was significantly lower than that of HF group (*P* < 0.05) (Fig. 1).

**Fig. 2:**
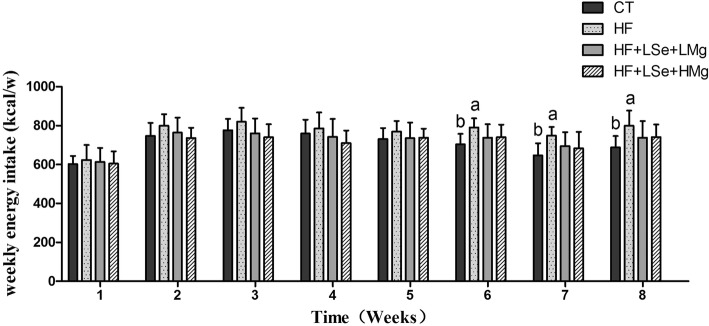
Effects of oral Se and Mg co administration on weekly energy intake in hyperlipidemia rats (n = 10, mean ± SD). a signify mean values that are significantly different from CT (*P* < 0.05). b signify mean values that are significantly different from HF (*P* < 0.05)

During the intervention period, the energy intake of the rats was not significantly different among the HF group and Se and Mg co-supplemented groups (*P* > 0.05). The energy intake of the HF group was greater than that of control group on 6 to 8 weeks (*P* < 0.05) (Fig. 2).

### Blood and liver lipids analyses

**Fig. 3:**
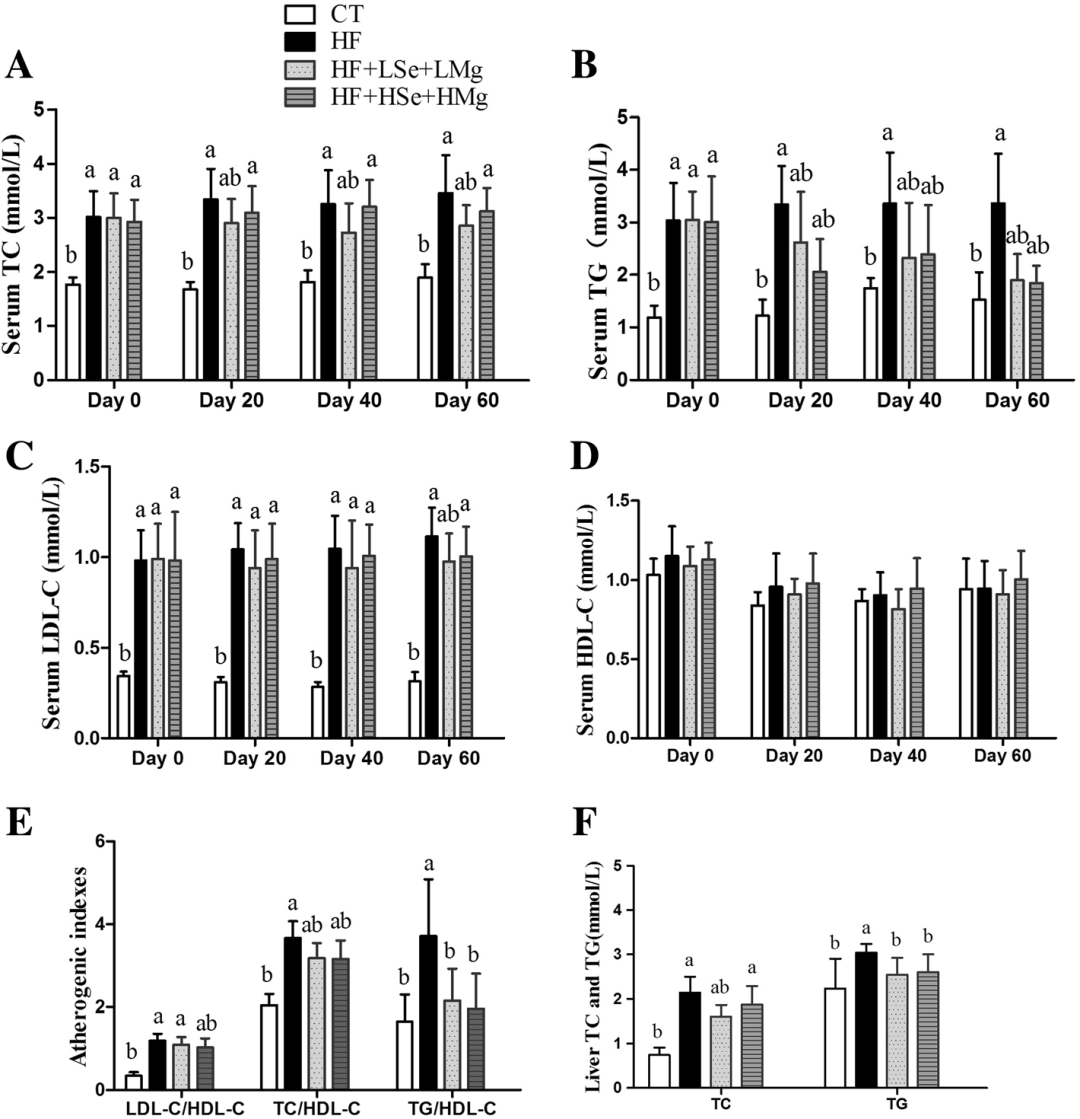
Effects of Se and Mg co administration on lipid metabolism in rats fed a high-fat diet. **a** Serum TC; **b** Serum TG; **c** Serum LDL-C; **d** Serum HDL-C; **e** Atherogenic indexes of the rats; **f** Liver TC and TG contents of the rats. The data are shown as the mean ± standard deviation (n = 10), a Significant difference with CT (*P* < 0.05). b Significant difference with HF (*P* < 0.05)

Serum TC, TG, LDL-C and HDL-C levels in the four groups were shown in Fig. 3a-d. On day 0 of the intervention, TC, LDL-C and TG showed no significant difference among HF, HF + LSe + LMg and HF+ HSe + HMg groups, but the rats in HF, HF + LSe + LMg and HF+ HSe + HMg groups fed a high-fat diet at the beginning of the study for 4 weeks had greatly increased levels of serum TC, LDL-C and TG levels compared to rats fed the normal diet (*P* < 0.05). This demonstrates that high-fat diet induced hyperlipidemic rats model was successful, which matched with the previous reports [19]. The elevated serum TC levels induced by feeding high-fat diets were significantly reduced in HF + LSe + LMg group on days 20, 40 and 60 compared to the HF group (*P* < 0.05) (Fig. 3a). The low dose supplementation of selenium and magnesium displayed in vivo hypocholesterolemic abilities. Serum TG level in HF + LSe + LMg and HF+ HSe + HMg groups on Day 20, 40 and 60 was significantly lower than that of HF group (*P* < 0.05) (Fig. 3b). Serum LDL-C level was significantly lower in HF + LSe + LMg group on Day 60 than that of HF group (*P* < 0.05) (Fig. 3c). There was no significant difference among all groups with respect to HDL in the present study. The high-fat diet had no obvious influences on the HDL-C level (Fig. 3d). The ratio of LDL-C/HDL-C in HF+ HSe + HMg group was significantly lower than that of HF group (*P* < 0.05), but it was still significantly higher than that of CT group (*P* < 0.05). The ratio of TC/HDL-C in HF + LSe + LMg and HF+ HSe + HMg groups was significantly lower than that of HF group (*P* < 0.05), but it was still significantly higher than that of CT group (*P* < 0.05). The ratio of TG/HDL-C in both HF + LSe + LMg group and HF+ HSe + HMg group was significantly lower than that of HF group (*P* < 0.05), and there was no significant difference compared with the CT group (*P* > 0.05) (Fig. 3e). Liver TC level of HF, HF + LSe + LMg and HF+ HSe + HMg groups was higher than that of CT group (*P* < 0.05), but which of HF + LSe + LMg group was lower than that of HF group (*P* < 0.05). For liver TG levels, there was significant difference between CT group and HF group (*P* < 0.05), and there was no significant difference in liver TG level between selenium and magnesium intervention groups and CT group (*P* > 0.05). Both HF + LSe + LMg and HF+ HSe + HMg groups had lower levels of liver TG than HF group (*P* < 0.05) (Fig. 3f).

### Clinical biochemistry

The blood biochemistry parameters of rats are shown in Table 3. On the 61th day of intervention, serum ALT and AST levels of the HF group were significantly higher than control group (*P* < 0.05), while serum ALT and AST level in both low-dose and high-dose selenium and magnesium co-supplementation groups were significantly lower than HF group (*P* < 0.05). TP and ALB levels in HF group and combined supplementation of selenium and magnesium intervention groups were significantly higher than those of control group (*P* < 0.05).

**Table 3:** Effects of oral Se and Mg co administration on blood biochemical index in hyperlipidemia rats

Group	Rats (n)	ALT (U/L)	AST (U/L)	TP (g/L)	ALB (g/L)	GLU (mmol/L)
CT	10	47.4 ± 3.6	146.4 ± 11.9	67.3 ± 5.0	43.4 ± 1.3	5.55 ± 0.31
HF	10	75.4 ± 9.4^a^	202.3 ± 44.5^a^	74.9 ± 6.1^a^	47.1 ± 4.5^a^	5.59 ± 0.64
HF + LSe + LMg	10	62.8 ± 18.7^ab^	138.2 ± 32.8^b^	73.4 ± 2.6^a^	46.0 ± 1.1^a^	5.61 ± 0.32
HF + HSe + HMg	10	53.4 ± 13.9^b^	156.3 ± 22.0^b^	73.9 ± 5.4^a^	46.9 ± 2.2^a^	6.08 ± 0.80

Values are expressed as mean ± SD of 10 rats per group. ^a^ Significant difference with CT (*P* < 0.05). ^b^ Significant difference with HF (*P* < 0.05). *TP* Total protein; *AST* Alanine aminotransferase; *ALT* Aspartate aminotransferase; *GLU* Glucose; *ALB* Albumin

### Effects of se and mg co-supplementation on the index of oxidation in the serum of rats

As shown in Table 4, the levels of SOD, GSH-Px, T-AOC did not have difference between CT and HF groups. The levels of serum antioxidant enzymes GSH-Px and SOD were significantly increased in HF + LSe + LMg and HF + HSe + HMg groups compared to CT and HF groups (*P* < 0.05). MDA level was significantly increased in serum of the HF group compared to the control group (*P* < 0.05). However, Se and Mg co-supplementation significantly lowered the MDA levels compared with the HF-treated group (*P* < 0.05). The groups which received co-administration of both the doses of Se and Mg showed a decrease of MDA and higher levels of GSH-Px and SOD.

**Table 4:** Effects of oral Se and Mg co administration on anti-oxidative enzymes in the serum oxidation of rats

Group	Rats (n)	T-AOC (U/L)	GSH-Px (U/mL)	SOD (U/mL)	MDA (μmol/L)
CT	10	8.89 ± 2.16	79.28 ± 37.64	41.65 ± 12.20	11.79 ± 4.98
HF	10	10.14 ± 1.50	80.79 ± 37.19	33.00 ± 10.12	25.03 ± 8.81^a^
HF + LSe + LMg	10	9.69 ± 3.95	154.01 ± 24.01^ab^	53.95 ± 9.49^ab^	16.56 ± 6.59^b^
HF + HSe + HMg	10	11.78 ± 2.27	140.84 ± 32.61^ab^	47.88 ± 15.28^b^	14.05 ± 4.60^b^

Values are expressed as mean ± SD of 10 rats per group. ^a^ Significant difference with CT (*P* < 0.05). ^b^ Significant difference with HF (*P* < 0.05). *GSH-Px* Glutathione peroxidase; *SOD* Superoxide dismutase; *T-AOC* Total antioxidative capacity; *MDA* Malondialdehyde

### Histopathology of liver

**Fig. 4:**
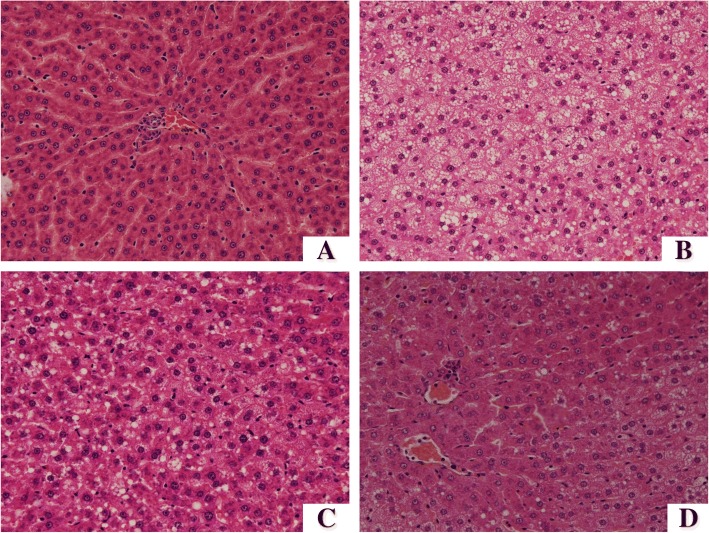
Histology of liver by hematoxylin and eosin staining (original magnification 400×). **a** control group; **b** model group; **c**: HF + LSe + LMg; D: HF + HSe + HMg

**Fig. 5:**
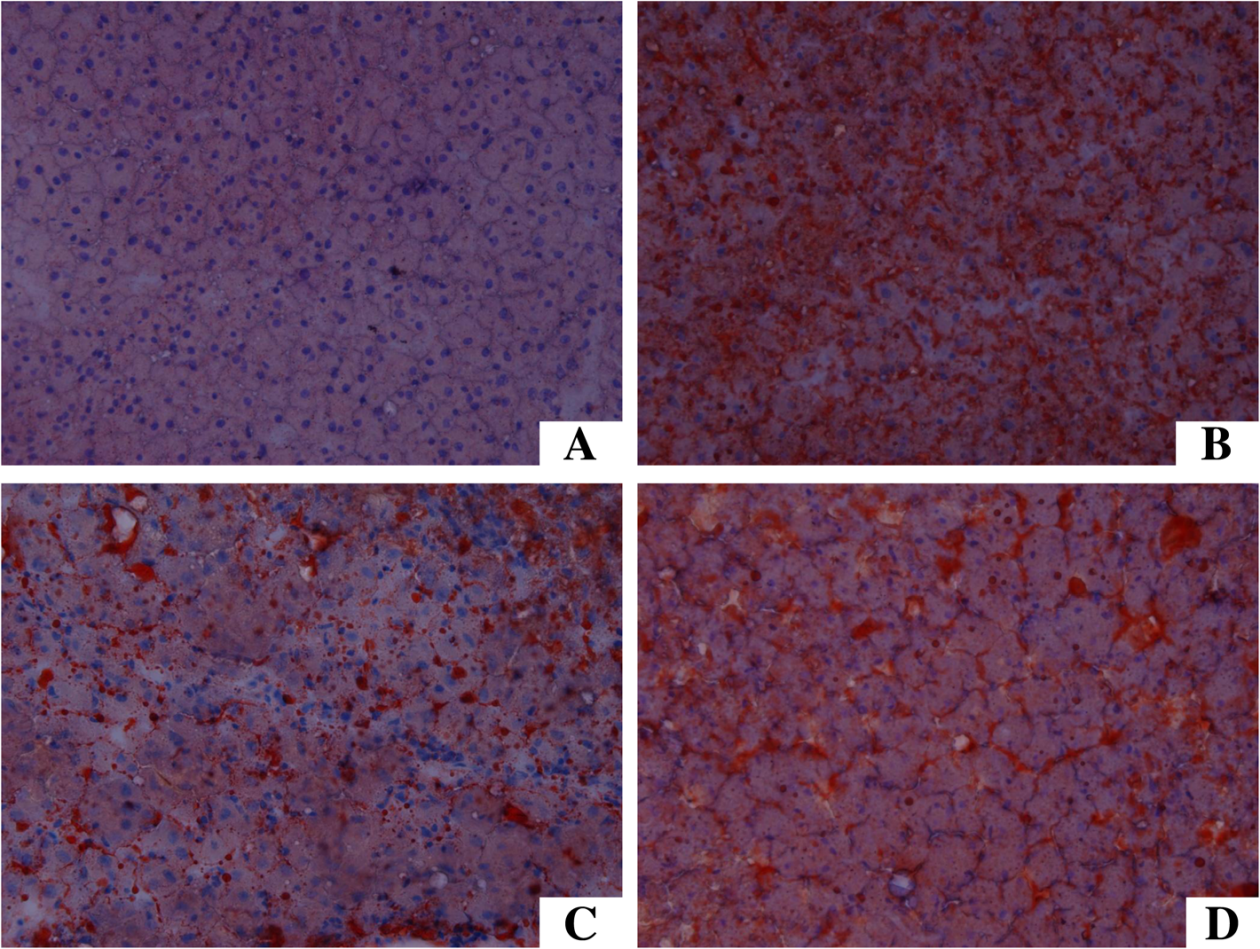
Histology of liver by Oil Red O staining (original magnification 200×). **a** control group; **b** model group; **c** HF + LSe + LMg; **d** HF + HSe + HMg

The liver tissue in the HF group had a moderate degree of vacuolization and increased lipid deposition in the cytoplasm, which was noticeably alleviated in the HF + LSe + LMg and HF + HSe + HMg groups (Fig. 4c and d). HF diet induced significant hepatic fat deposits in rats compared to control group (Fig. 5a and b). Oil red O staining showed multiple massive lipid droplets were accumulated in the liver tissues in HF group and the lipid droplets were reduced in HF + LSe + LMg and HF + HSe + HMg groups (Fig. 5).

### mRNA expression levels of liver enzymes associated with lipid metabolism

**Fig. 6:**
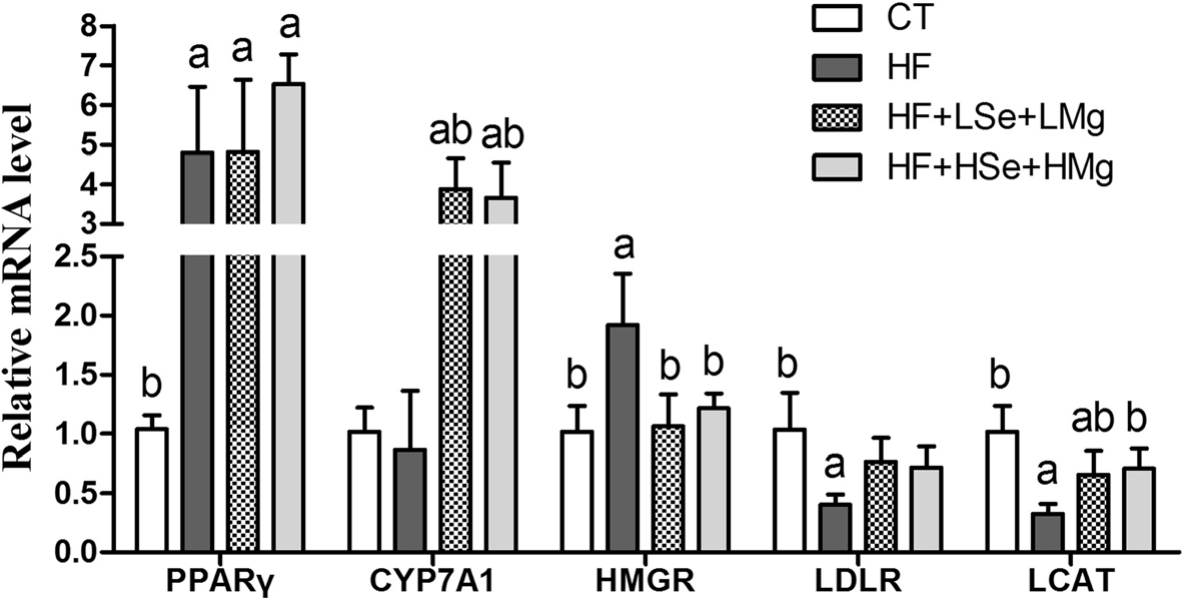
Effects of the Se and Mg co administration on mRNA expression levels of liver enzymes associated with cholesterol metabolism in rats fed a high-fat diet. Gene expression levels are expressed as values relative to the control group. **a** Significant difference with Control (*P* < 0.05). **b** Significant difference with HF (*P* < 0.05)

**Fig. 7:**
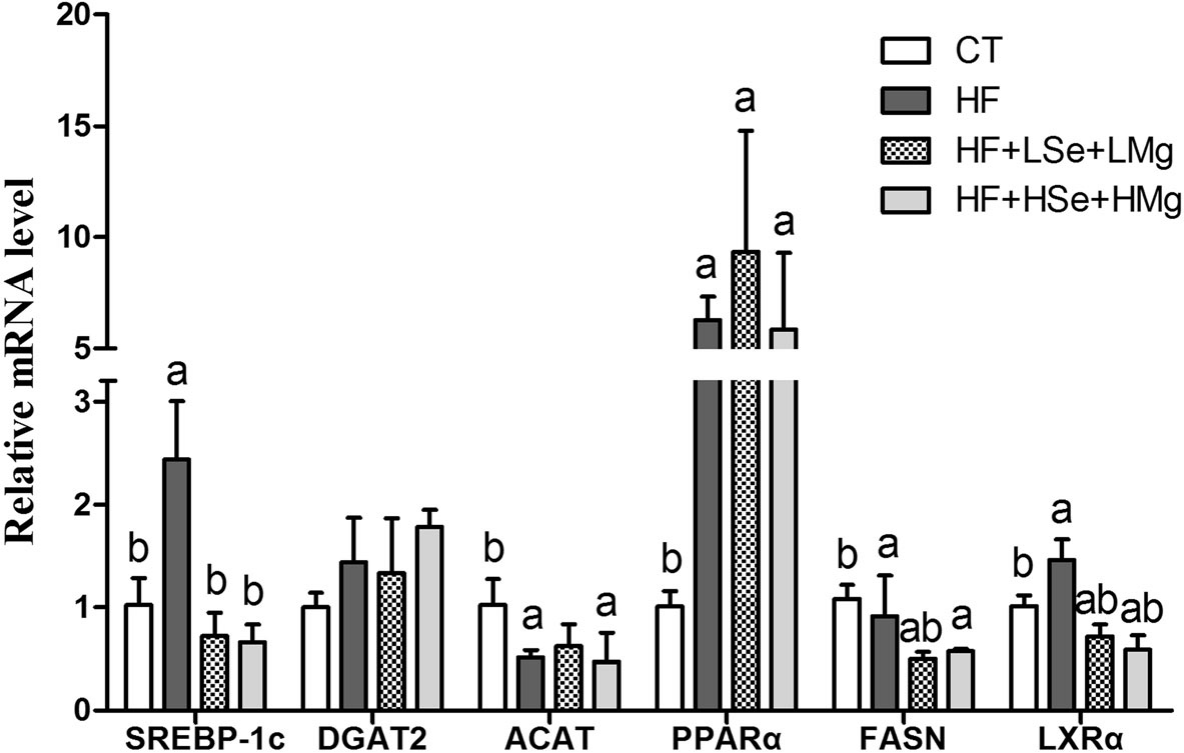
Effects of the Se and Mg co administration on mRNA expression levels of liver enzymes associated with fatty acids and TG (synthesis/metabolism) in rats fed a high-fat diet. Gene expression levels are expressed as values relative to the control group. **a** Significant difference with Control (*P* < 0.05). **b** Significant difference with HF (*P* < 0.05)

The mRNA expression levels of hepatic enzymes associated with lipid metabolism are illustrated in Figs. 6 and 7. Cholesterol 7α-hydroxylase (CYP7A1) is the key enzyme in the synthesis of bile acid from cholesterol. The levels of expression of its mRNA in HF + LSe + LMg and HF + HSe + HMg groups were significantly increased compared to the HF group (*P* < 0.05), 4.51- and 4.26-fold higher than HF group respectively. 3-hydroxy-3-methyl glutaryl coenzyme A reductase (HMGR) is a rate-limiting enzyme for cholesterol synthesis in vivo. The decrease of its activity contributes to the reduction of cholesterol synthesis [20]. Lecithin cholesterol acyltransferase (LCAT) is a key enzyme in the metabolism of cholesterol, and plays an important role in the cholesterol esterification and metabolism of HDL, LDL and TG. LCAT plays an antioxidant role in vivo and affects lipid metabolism [21]. HF feeding decreased cholesterol metabolism gene mRNA expression of lecithin cholesterol acyltransferase (LCAT), and increased cholesterol synthesis speed limiting enzyme HMGR and peroxisome proliferator-activated receptor gamma (PPARγ). We found that Se and Mg co-supplementation significantly reversed HF gene expression changes including up-regulation of cholesterol esterification enzyme (LCAT) and down-regulation of endogenous cholesterol synthesis enzyme (HMGR) (*P* < 0.05) (Fig. 6). Sterol regulatory element-binding protein-1c (SREBP-1c) regulates the synthesis of fat by activating genes involved in fatty acids and triglycerides. Genes involved in the regulation of fatty acids and TG synthesis/metabolism showed significantly increased levels of LXRα, SREBP-1c and FASN in the HF fed group compared to the control group(*P* < 0.05). The groups which received both doses of Se and Mg co-supplementation showed decreased their levels (*P* < 0.05) (Fig. 7).

## Discussion

Studies have shown that high fat diets can provoke dyslipidemia in rodents and promote lipid synthesis-related gene expression (such as SREBP-1c and HMGR) [22]. In this study, the rats fed HF showed highly significant levels of total cholesterol as well as triglycerides in the serum and liver when compared to the control rats. Co-supplementation of selenium and magnesium led to a significant reduction in the levels of these parameters *vis-a-vis* HF fed animals in both serum and liver. Low dose of selenium and magnesium supplementation significantly reduced the elevated levels of serum and liver TC and serum LDL-C induced by feeding high-fat diets, however, high doses had no significant effect on lowering blood TC and LDL-C and liver TC. It is presumed that the high dose Se of 100 μg/kg BW is probably beyond the sensitive dose range of selenium that can affect the blood and liver lipid levels. So the low dose supplementation of selenium and magnesium displayed better in vivo hypocholesterolemic abilities.

Previous studies showed that administration of magnesium (100 mg Mg/l water) together with selenium (0.4 mg Se/l water) significantly enhances antioxidant defences against alcohol-induced oxidative stress, disturbance of liver function and cholesterol metabolism [23]. In this study, the activity of serum antioxidant enzymes GSH-Px and SOD was significantly increased in both doses of selenium & magnesium supplementation groups compared to HF group. The main functions of selenium in the organism are connected with its antioxidant properties, as it is an essential cofactor of important antioxidant enzymes [24]. Magnesium is an important component of the antioxidant system and may be used as potential therapeutic agent to reduce the clinical diseases associated with increased oxidative stress. Hans et al. [25] has reported that magnesium treatment helps to restore the hepatic activity of glutathione S-transferase (GST) and superoxide dismutase (SOD), and reduces oxidative stress in diabetic rats. MDA is one of the final products of polyunsaturated fatty acids peroxidation in the cells. An increase in free radicals causes overproduction of MDA. MDA levels are commonly known as a marker of oxidative stress and the antioxidant status, as well as CVD risk [26]. In the present study, MDA increased in the model compared with the control group, but selenium and magnesium co-supplementation reduced serum MDA levels.

Selenium and Mg co-supplementation administration for two months ameliorated HF-induced hepatic steatosis and liver dysfunction in rats, as indicated by plasma parameters and Oil Red O staining. The effect of hypolipidemic of dietary magnesium may be due to the ability of magnesium to reduce the activity of lipogenic enzymes or raise the activity of lipoprotein lipase [27]. Sreekala et al. [28] investigated the effect of sodium selenite on hyperlipidemia rats induced by nicotine, and showed that while an intervention dose of 1 μg Se/Kg and 50 μg Se/Kg Body WT could both reduce high cholesterol levels induced by nicotine, the selenium dose of 1 μg Se/Kg was more effective with high doses of selenium causing liver toxicity. Low dose sodium selenite (1 μg Se/Kg Body WT) significantly reduced lipogenic enzyme and HMG-CoA reductase (HMGR) activity. Dhingra et al. reported that when supplementation of selenium diet reached 1 ppm for three months, that dose could increase LDL receptor (LDLR) activity and mRNA expression [29], and reduce cholesterol, LDL-C and ApoB by down regulating HMGR mRNA expression in hypercholesterolemic rats [30]. In our study, HF feeding decreased cholesterol metabolism gene mRNA expression of lecithin cholesterol acyltransferase (LCAT), and increased cholesterol synthesis speed limiting enzyme HMGR and peroxisome proliferator-activated receptor gamma (PPARγ), whereas Se and Mg co-supplementation significantly increased the levels of expression of LCAT mRNA and decreased that of HMGR (*P* < 0.05). CYP7A1 promotes the metabolism of cholesterol into bile acids. Over-expression of the mRNA level of CYP7A1 significantly decreased the serum level of TC and LDL-C in hamsters fed a high-fat diet [31]. In our study, the mRNA level of liver CYP7A1 was increased by Se and Mg supplementation by gavage than fed high-fat diet alone. The mechanism of Se combined with Mg in regulating cholesterol metabolism may be achieved by reducing cholesterol endogenous synthesis (HMGR) and enhancing the transport of cholesterol into hepatocyte (CYP7A1) and cholesterol esterification (LCAT). Sterol regulatory element-binding protein-1c (SREBP-1c) is the key regulator of lipid metabolism and is sensitive to nutritional status. Activation of SREBP-1c increases hepatic lipogenesis under high dietary conditions and leads to fatty liver [32]. The expression levels of SREBP-1c mRNA were increased in the high-fat diet fed group, but both doses of Se and Mg co-supplementation groups significantly decreased its levels. LXRα is a key enzyme gene for fatty acid production, and FASN is its downstream gene. LXRα regulates the key enzyme of fat synthesis, which can regulate the expression of FASN by directly combining with FAS promoter or indirectly through the SREBP-1c pathway to up regulate the expression of FASN [33]. Over expression of SREBP-1c can induce increased transcription of FASN, resulting in increased fatty acid synthesis. Increase of fatty acid synthesis leads to abnormal deposition of fat in hepatocyte then leads to fatty liver formation. Our results support such a mechanism, showing that suppression of SREBP-1c expression by Se and Mg co-supplementation is mediated by stimulation of LXRα expression. Taken together, Se and Mg co-supplementation regulate hepatic genes responsible for de novo fatty acid synthesis via modulation of LXRα/SREBP-1c pathway and diminishes HF-induced fatty liver and hyperlipidemia.

## Conclusions

To the best of our knowledge, the present contribution is that it is the first time that selenium combined with magnesium in the treatment of hyperlipidemia is investigate. Combination of Se and Mg exerted significant antihyperlipidemic effects and reduced hepatic lipids in rats fed a high-fat diet, with low dose Se and Mg co-supplementation trending towards more significant hypocholesterolemic effects. Co-supplementation of selenium and magnesium can effectively regulate abnormal lipid metabolism, correct hyperlipidemia and fatty liver, suggesting that selenium and magnesium co-supplementation may be a potential nutraceutial in overcoming the negative effects of lipid disorders beyond pharmacological interventions, for ameliorating hyperlipidemia and improving antioxidant capacity. Se combined with Mg in reducing serum TC, LDL-C and liver TC may be achieved by reducing cholesterol endogenous synthesis (HMGR) and enhancing the transport of cholesterol into hepatocyte (CYP7A1) and cholesterol esterification (LCAT). Se and Mg co-supplementation regulated hepatic genes responsible for de novo fatty acid synthesis via modulation of LXRα/SREBP-1c pathway and diminishes HF-induced fatty liver and hyperlipidemia.

## Abbreviations

ACAT: Acyl coenzyme A–cholesterol acyltransferase
Acyl CoA: Acyl coenzyme A
ALB: Albumin
ALT: Aspartate aminotransferase
AST: Alanine aminotransferase
CYP7A1: Cholesterol 7α-hydroxylase
DGAT2: Diacylgycerol acyltransferase 2
FASN: Fatty acid synthase
GLU: Glucose
GSH-Px: Glutathione peroxidase
HDL-C: High-density lipoprotein cholesterol
HE: Hematoxylin-eosin
HMG-CoA-R: 3-hydroxy-3-methyl glutaryl coenzyme A reductase
LCAT: Lecithin cholesterol acyltransferase
LDL-C: Low-density lipoprotein cholesterol
LDLR: Low-density lipoprotein receptor
LXRα: Liver X receptor alpha
MDA: Malondialdehyde
PPARα: Peroxisome proliferator-activated receptor alpha
PPARγ: Peroxisome proliferator-activated receptor gamma
SD: Standard deviations
SOD: Superoxide dismutase
SREBP-1c: Sterol regulatory element-binding proteins
T-AOC: Total antioxidative capacity
TC: Total-cholesterol
TG: triglycerides
TP: Total protein
